# Temporal transcriptomics provides insights into host‒pathogen interactions: a case study of *Didymella pinodella* and disease-resistant and disease-susceptible pea varieties

**DOI:** 10.1007/s44297-023-00005-w

**Published:** 2023-08-10

**Authors:** Chao Liu, Xingmin Han, Jacob L. Steenwyk, Xing-Xing Shen

**Affiliations:** 1 Key Laboratory of Biology of Crop Pathogens and Insects of Zhejiang Province, College of Agriculture and Biotechnology and Centre for Evolutionary & Organismal Biology, Zhejiang University, Hangzhou, 310058 China; 2 Zhejiang Lab, Hangzhou, 311121 China; 3 Howards Hughes Medical Institute and the Department of Molecular and Cell Biology, University of California, Berkeley, Berkeley, CA 94720 USA

**Keywords:** *Didymella pinodella*, Pea, Genome sequencing, Comparative genomics, Comparative transcriptomics, Pathogenicity, Infection, Defense, Interactions

## Abstract

Ascochyta blight is a fungal disease affecting peas, causing significant damage to the plant and reducing crop yield. Host‒pathogen interactions can inform disease prevention and control strategies but remain poorly understood. Here, we generate a near-chromosome-level assembly for *Didymella pinodella* HNA18, a pathogenic fungus that causes pea ascochyta blight. Comparative genomic analysis of *D. pinodella* HNA18 and seven publicly available *Didymella* genomes revealed that the genome of *D. pinodella* HNA18 encodes the most conserved biosynthetic gene clusters (BGCs) and a similar number of carbohydrate-activating enzyme (CAZyme) genes compared to other *Didymella* species. Furthermore, by sequencing and analyzing the transcriptomic data of *D. pinodella* HNA18 and disease-susceptible and disease-resistant pea varieties during the infection process, we found that the pathogenic fungus mobilized a similar set of infection genes to attack the disease-susceptible and disease-resistant pea varieties, but the timing and intensity of these infection genes were different. For pea varieties in response to the pathogenic fungus, disease-susceptible and disease-resistant pea varieties mobilized similar types of defense genes, while the disease-resistant pea used a higher number of defense genes relative to the disease-susceptible pea during the entire infection process. This study not only provides multiomic resources for the study of the pathogenic fungus *D. pinodella* HNA18 against its disease-susceptible and disease-resistant pea varieties but also deciphers the mode of interaction between pathogenic fungal infection and plant defense.

**Supplementary Information:**

The online version contains supplementary material available at 10.1007/s44297-023-00005-w.

## Introduction

Plant pathogenic fungi negatively impact the global food supply by reducing crop yield, quality, and marketability of staple foods such as wheat, rice, and peas [1, 2]. Plant pathogenic fungi may also produce toxins, which not only facilitate disease progression but also pose a threat to human health [3, 4]. Ascochyta blight, or fungal leaf spot, is a serious disease of peas, reducing crop yield by 28–88%, often caused by *Didymella pinodella* [5–7]. Despite the burden *Didymella* species pose, little has been done to characterize their genomic content, in part owing to the paucity of available genome sequences. Moreover, host‒pathogen interactions are understudied in *Didymella* pathogens.

To date, there are three main types of studies that have examined the interaction between pathogenic fungi and host plants: 1) A pathogenic fungus infects different plant species. For example, when *Fusarium oxysporum* infects multiple cash crops (e.g., wheat, tomatoes and bananas), several plant-dependent resistance genes have been identified [8]. 2) Different pathogenic fungi infect a specific plant. For example, *Fusarium graminearum* and *Magnaporthe oryzae* are both important cereal pathogenic fungi, in which both pathogenic fungi can trigger similar defense pathways in the host plant *Brachypodium distachyon* but exhibit different expression programs and regulations of host defense genes [9]. 3) Different strains of the same pathogenic fungus infect the same plant. For example, different strains of *Cercospora sojina* infected soybean and exhibited varying pathogenicity. Specifically, the strongly pathogenic strain significantly increased the expression of virulence-related genes and carbohydrate-activating enzyme (CAZyme) genes, which are essential for pathogenic fungi penetrating the plant cell wall and growth [10], compared to the weakly pathogenic strain [11]. Although these studies have greatly enriched our understanding of the strategies of either pathogenic fungi or host plants, the pattern of simultaneous interactions between pathogenic infection and plant defense using the same pathogenic fungus against different varieties of the same host plant species remains poorly understood.

One strategy to decrease the burden of plant pathogenic fungi is disease-resistance breeding among crops. For example, disease-resistant pea varieties have been bred by crossing with wild peas [12–14]. However, disease-resistant varieties may exhibit slower growth [15] due to competitive costs between growth and defense [16]. Other differences between resistant and susceptible crops remain underexplored—for example, the transcriptional response between disease-susceptible and disease-resistant cultivars across stages of infection. Transcriptomics during infection provides an opportunity to expand our understanding of host‒pathogen interactions and identify genes putatively associated with host defense and microbial pathogenicity [9, 17].

Here, we generate a near-chromosome-level assembly of *Didymella pinodella* HNA18, a pathogenic fungus of pea ascochyta blight, and shed light on the genetic and transcriptional underpinnings of host‒pathogen interactions in disease-resistant and disease-susceptible cultivars of pea (*Pisum sativum*). Comparative genomics of *Didymella pinodella* HNA18 with seven other *Didymella* species revealed that the genomes encode many carbohydrate-activating enzyme (CAZyme) genes and secondary metabolic gene clusters (SMGCs). Transcriptomic profiling of *D. pinodella* HNA18 and the disease-resistant and disease-susceptible cultivars of pea across three timepoints revealed an interplay between pathogenic infection genes and plant defense genes wherein the pathogenic fungus mobilized a similar set of infection genes to attack the disease-susceptible and disease-resistant pea varieties, but the timing and intensity of these infection genes were different, and disease-susceptible and disease-resistant pea varieties mobilized similar types of defense genes, while the disease-resistant pea used a higher number of defense genes relative to the disease-susceptible pea. This study provides important multiomic resources for the study of the pathogenic fungus *D. pinodella* HNA18 against its disease-susceptible and disease-resistant pea varieties and a new perspective for the study of the interaction pattern between pathogenic fungi and plants.

## Results

### *Didymella pinodella* HNA18 was identified as the fungal pathogen causing black rot of pea

**Fig. 1:**
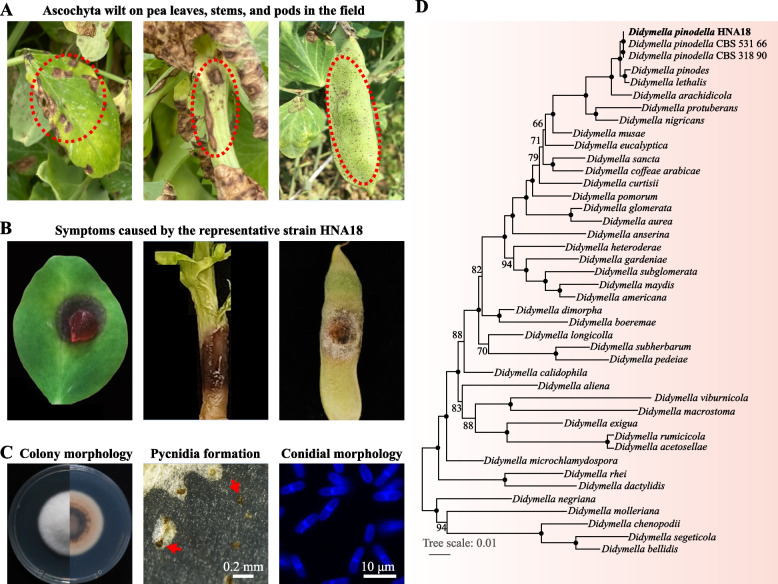
Symptoms of ascochyta blight on pea and identification of the pathogenic strain HNA18. **A** Leaf, stem, and pod lesions of ascochyta blight on pea collected in the field. **B** Symptoms caused by strain HNA18 under laboratory conditions. **C** Morphological characteristics of the strain HNA18. From left to right: colony of HNA18 on CM, pycnidia formation of HNA18 on CM, and conidial morphology of HNA18. The typical pycnidia are indicated by red arrows. Conidia were stained with calcofluor white. **D** Phylogenetic tree constructed using the maximum likelihood method based on four widely used loci (LSU, TUB, ITS and RPB2). Branch support values near internodes/internal branches correspond to ultrafast bootstrap support. Only support values smaller than 95% are shown

Ascochyta blight is a serious disease that causes brown spots on pea leaves and pods [5, 6, 18]. Severe infections can cause brown discoloration and streaks on the lower part of the stem. We isolated two strains of filamentous fungi, HNA18 and HB8, from the cultures of diseased pea tissue. After reinoculation of pea leaves, only HNA18 caused ascochyta blight (Fig. 1A). After inoculating peas with HNA18 for 3 days, disease symptoms—such as darkening of the inoculated spot and waterlogged rot—were identified along the pea leaf, stem, and pod (Fig. 1B), suggesting that the etiological agent of disease had successfully been isolated. Morphological characterization of HNA18 in standard laboratory conditions revealed that colonies were white, round, with flocculent, dense mycelium when grown on complete medium (CM) for 6 days (Fig. 1C); colonies turned white and brown after 15 days. Asexual fruiting bodies (pycnidia) begin to form after approximately 10 days of incubation (Fig. 1C). Conidia were typically oval to long oval and had one septum (Fig. 1C). These structures are typically observed in fungi; however, molecular techniques are required to determine the exact genus and species. To this end, comparison of the ITS (internally transcribed spacer) sequence from HNA18 genomic DNA against NT (nucleotide sequence database) using BLAST revealed 100% sequence similarity with *Didymella pinodella*. Molecular phylogenetics of 40 *Didymella* species and two strains of *D. pinodella* using four taxonomically informative loci (large subunit, β-tubulin, internally transcribed spacer, and RNA polymerase second largest subunit) revealed that strain HNA18 was nested within other strains of *Didymella pinodella* with 100% bootstrap support (Fig. 1D). Taken together, these results provide robust support that *Didymella pinodella* HNA18 was the isolated pathogen causing black rot in the pea.

### A near-chromosome level assembly of *Didymella pinodella* HNA18

**Fig. 2:**
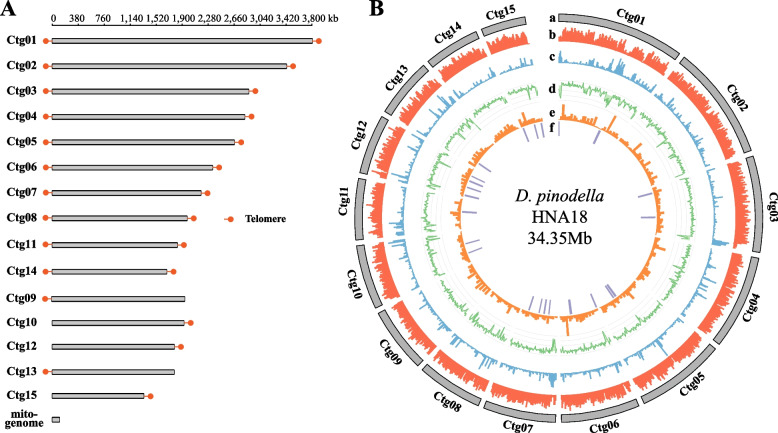
Overview of the genome of *D. pinodella* HNA18. **A** Genome contigs of *D. pinodella* HNA18 were assembled using PacBio long reads along with Illumina short reads. Fifteen nuclear contigs are labeled from Ctg01 to Ctg15. The mitochondrial genome was also included at the bottom. Telomeres for each of 15 nuclear contigs are colored by red dots (see Materials and methods). **B** CIRCOS plot of the genomic features of the *D. pinodella* HNA18 reference genome. Tracks labeled from outside to inside: (a) The 15 contigs of the *D. pinodella* HNA18 genome; (b) The density of genes in the genome in 25 kb windows; (c) The density of repeat sequences in the genome in a 25 kb window. (d) The difference between the GC content and the average GC content of the genome; (e) The coverage of RNA-seq reads on the genome; (f) The location of the secondary metabolic synthesis gene cluster on the genome

A combination of long- and short-read technologies was used to obtain a chromosome-level assembly of the *D. pinodella* HNA18 genome. Specifically, the genome was assembled into 15 contigs (total size = 34.35 Mb, N50 = 2.34 Mb, and GC content = 50%; Table 1) using 571,275 quality-filtered PacBio reads (average length = 11,998 base pairs). The PacBio average read-depth coverage was 199.5. A complete mitochondrial genome 73,728 bp in length was also assembled. Genome quality assessment showed that the genome assembly of *D. pinodella* HNA18 contained 3,769/3,786 (99.55%) out of all 3,786 near-universally single-copy orthologs encoded in the genomes of fungi from the class *Dothideomycetes* [19]. The telomeric repeats “TTAGGG” have been identified in other filamentous fungi—*Neurospora crassa* [20], *Cladosporium fulvum* [21] and *Magnaporthe oryzae* [22, 23]—and were queried against the genome assembly. Among 15 contigs, ten had “TTAGGG” repeats flanking both contig ends, and five had “TTAGGG” repeats at one end (Fig. 2A). Transcriptome-guided gene annotation of the *D. pinodella* HNA18 genome resulted in 11,201 putative genes—11,019 protein-coding genes, 140 tRNAs, and 42 rRNAs—with an average gene density of 326 genes per Mb (Fig. 2B and Table 1).

**Table 1:** The features of the genome assembly of *Didymella pinodella* HNA18

**Features**	**Values**
PacBio sequence reads	571,275
Genome size (bp)	34,354,111
Number of contigs	15
The Largest contig (bp)	3,802,609
Contig N50 (bp)	2,342,718
GC content	52.65%
Coverage depth	199.5X
Number of complete BUSCOs	3,769 (99.5%)
Number of fragmented BUSCOs	4 (0.1%)
Number of missing BUSCOs	13 (0.4%)
Number of putative genes	11,201
Number of protein-coding genes	11,019
Gene density (no. genes/1 Mb)	326
Number of rRNA	42
Number of tRNA	140
The percentage of repeats in genome assembly	10.48%
Mitochondrial genome size (bp)	73,728
Number of secondary metabolic gene clusters	24
Number of CAZyes genes	593

### Genetic diversity of gene families, CAZymes, and secondary metabolic gene clusters

**Fig. 3:**
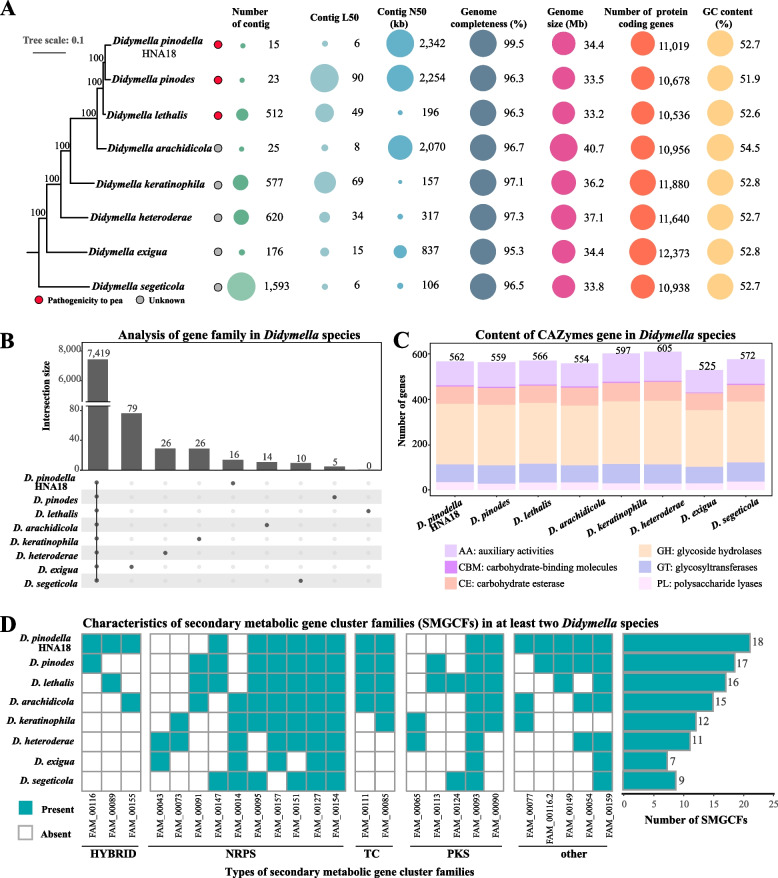
Comparative genomic analysis of *D. pinodella* HNA18 and seven other *Didymella* species. **A** A phylogenetic tree was constructed based on maximum likelihood using 5,699 single-copy genes. The circles at the end of the branches represent the pathogenicity of the eight *Didymella* species. The red circles denote the ability to cause pea disease; the gray circles denote unknown pathogenicity information. All pathogenicity information for the eight *Didymella* species is also given in Supplementary Table 1. Eight bubble plots show key genome qualities and features. The bubble sizes have been proportionally scaled for each panel. **B** Number of gene families shared among the eight *Didymella* species and number of species-specific gene families. **C** The total number of carbohydrate-activating enzyme (CAZyme) genes in each *Didymella* species. The CAZyme genes were assigned to six categories: AA, CBM, CE, GH, GT, and PL. **D** Analysis of the secondary metabolite gene clusters (SMGCs) in the eight *Didymella* species. The matrix in the left section represents the presence and absence of SMGC families. The bars in the right section represent the total number of SMGC families that were found in two or more species. HYBRID: a backbone gene containing domains from NRPS and PKS backbones, NRPS: nonribosomal peptide synthetase, PKS: polyketide synthase, TC: terpene cyclase

To examine the features of the *D. pinodella* HNA18 genome and seven other publicly available *Didymella* species genomes, we first constructed a genome-scale phylogenetic tree of the genus *Didymella* using 5,699 single-copy genes. The phylogenetic tree showed that all internodes received 100% bootstrap support values, and *D. pinodella* HNA18 was the sister to *D. pinodes* (Fig. 3A). Examination of metrics of genome assembly quality revealed that *D. pinodella* HNA18 had the most contiguous assembly, with the smallest number of contigs (15), the lowest contig L50 (6), the longest contig N50 (2.34 Mb), and the highest genomic completeness (99.55%) (Fig. 3A). Comparison of genome size, number of protein-coding genes, and GC content revealed similar features across *Didymella* species; specifically, genomes ranged from ~33–40 Mb, encoded ~11,000–12,000 genes, and had GC contents of ~52–55%.

To assess the conservation and diversity of the gene content in the eight *Didymella* genomes, we clustered the 90,020 protein-coding genes from eight *Didymella* species genomes into putative gene families. This analysis showed that 7,419/11,980 (62%) gene families were shared between the eight *Didymella* species. A total of 176 gene families were species specific; 16 of 176 were specific to HNA18 (Fig. 3B and Supplementary Table 1). Given that the total number of genes in the genomes of the eight *Didymella* species is 87,594, 73.69% (64,549/87,594) of the protein-coding genes are evolutionarily conserved throughout the genus *Didymella*.

Carbohydrate-activating enzymes (CAZymes) play a major role in fungal degradation of plant polysaccharides and are associated with fungal plant pathogenicity [24]. The eight *Didymella* species harbored CAZyme genes ranging from 525 to 605, with an average of 567 (Fig. 3C). The number of identified CAZyme genes in eight *Didymella* species was consistently higher than that (475 CAZyme genes) in pathogenic fungi from a previous study [24].

The genomes of plant pathogenic fungi can also encode SMGCs, which are responsible for the biosynthesis of toxic molecules that can facilitate disease progression and threaten human health if ingested through infected crops [25]. To determine the secondary metabolic potential of each *Didymella* species, SMGCs were identified in each *Didymella* genome. The number of SMGCs ranged from 14 to 29 in the eight *Didymella* species (Supplementary Figure 1). Three SMGCs were present in all species (Fig. 3D), and 25 SMGCs were present in at least two species. The genome of *D. pinodella* HNA18 encoded the highest number of SMGCs present in at least two species (18) (Fig. 3D). Interestingly, we found that FAM_00091 (NRPS) is present in *D. pinodes*, *D. lethalis*, and *D. arachidicola* but is absent in *D. pinodella* HNA18. The FAM_00091 (NRPS) gene cluster exhibits diverse functions, including the synthesis of bioactive substances and pigments [26]. The loss of FAM_00091 in *D. pinodella* HNA18 might be involved in functional differences in the synthesis of bioactive substances and pigments.

### Host‒pathogen transcriptomics maps disease progression in disease-susceptible and disease-resistant pea varieties

**Fig. 4:**
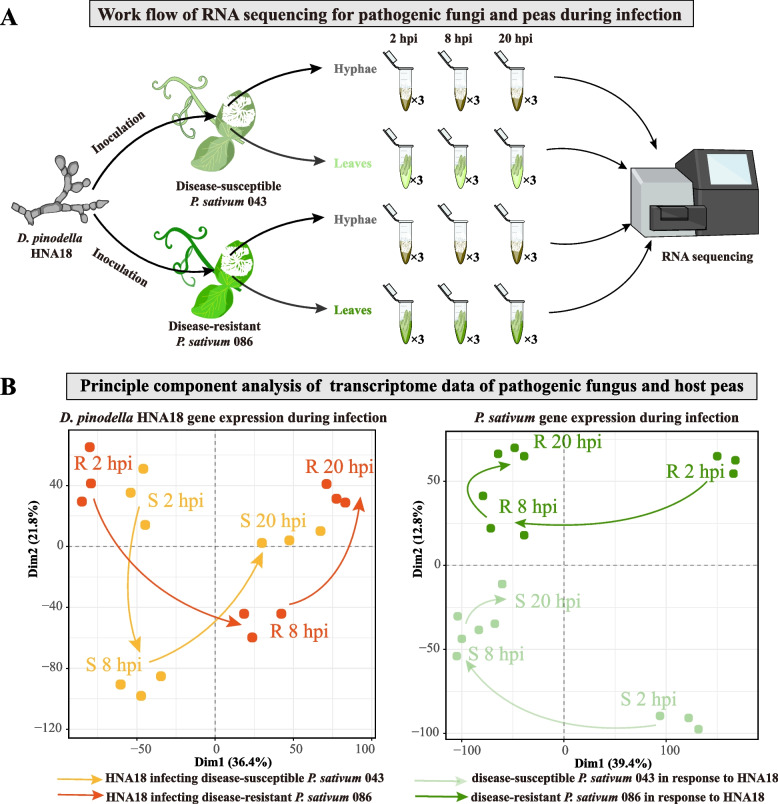
Transcriptome sampling and sequencing of the pathogenic fungus and disease-susceptible and disease-resistant pea varieties. **A ***D. pinodella* HNA18 mycelium was inoculated on leaves of disease-susceptible *P. sativum* 043 and disease-resistant *P. sativum* 086. The fungal mycelium and pea leaves were separately sampled for RNA sequencing at 2 h postinfection (2 hpi), 8 h postinfection (8 hpi), and 20 h postinfection (20 hpi). Each sample had three replicates. **B** Principal component analysis of transcriptomic data of *D. pinodella* HNA18 and disease-susceptible and disease-resistant pea varieties during three infection stages. Arrows indicate progression through the real-time

To gain insight into changes in the transcriptome profiles of the host and pathogen during disease progression, we conducted RNA sequencing of pathogen hyphae and host leaves in disease-susceptible and disease-resistant *P. sativum* varieties (043 and 086, respectively) across disease progression (Fig. 4A). Infection was caused by inoculating leaves with fungal mycelium. Samples were collected during the contact (2 hpi), penetration (8 hpi), and lesion formation stages (20 hpi) (Fig. 4A) with three replicates for each stage. The resulting 36 samples were sequenced using Illumina HiSeq2000 (pair ends), generating approximately 883 million pairs of quality-filtered 150 base pair reads (Supplementary Table 2). Reads from the fungal and host pea samples were mapped to the *D. pinodella* HNA18 and *P. sativum* v1a genomes, respectively.

Principal component analysis (PCA) of host and pathogen gene expression can offer insight into the infection process [27]. For the transcriptomic profiles of *D. pinodella* HNA18, dimensions 1 (36.4% of the variance) and 2 (21.8% of the variance) captured the majority of the transcriptional variance induced during infection (Fig. 4B). At 2 hpi and 20 hpi, pathogen transcriptomic profiles were similar in disease-susceptible and disease-resistant pea hosts. In contrast, at 8 hpi, the transcriptomic profile of the pathogen was different in disease-susceptible and disease-resistant hosts. For the transcriptomic profiles of the host pea, PCA revealed that disease-susceptible and disease-resistant pea varieties exhibited substantially different transcriptomic profiles in response to *D. pinodella* HNA18 infections at all stages of disease along the second dimension (12.8% of the variance) but were similar along the first dimension, which captured the majority of variance (39.4% of the variance).

### Differential expression analysis implicates plant polysaccharide degradation genes in infection

To determine how transcriptional responses change throughout disease progression, we identified differentially expressed genes in the pathogen (*D. pinodella* HNA18) and host (disease-susceptible and disease-resistant *P. sativum* varieties) by comparing 8 hpi with 2 hpi and 20 hpi with 2 hpi. These analyses revealed that *D. pinodella* HNA18 differentially expressed 1,644 genes (1,141 up- and 503 downregulated) when infecting disease-susceptible *P. sativum* 043 at 8 hpi and 3,420 differentially expressed genes (1,949 up- and 1,471 downregulated) when infecting disease-resistant *P. sativum* 086 at 8 hpi (Supplementary Figure 2A and Supplementary Table 3). In contrast, similar numbers of differentially expressed genes in *D. pinodella* HNA18 were observed at 20 hpi (Supplementary Figure 2B and Supplementary Table 3); specifically, there were 2,972 differentially expressed genes (1,646 upregulated and 1,326 downregulated) and 4,066 differentially expressed genes (2,153 upregulated and 1,913 downregulated) when infecting disease-susceptible and -resistant *P. sativum* varieties at 20 hpi, respectively (Supplementary Figure 2B and Supplementary Table 3). Comparing all upregulated differentially expressed genes in *D. pinodella* HNA18 across all stages of disease progression revealed that 1,802 genes were upregulated in the disease-susceptible and disease-resistant pea varieties (1,802/2,144 [84%] and 1,802/2,691 [67%], respectively). These 1,802 genes may provide insight into the transcriptional underpinnings of disease caused by *D. pinodella* in both disease-resistant and disease-susceptible pea varieties.

**Fig. 5:**
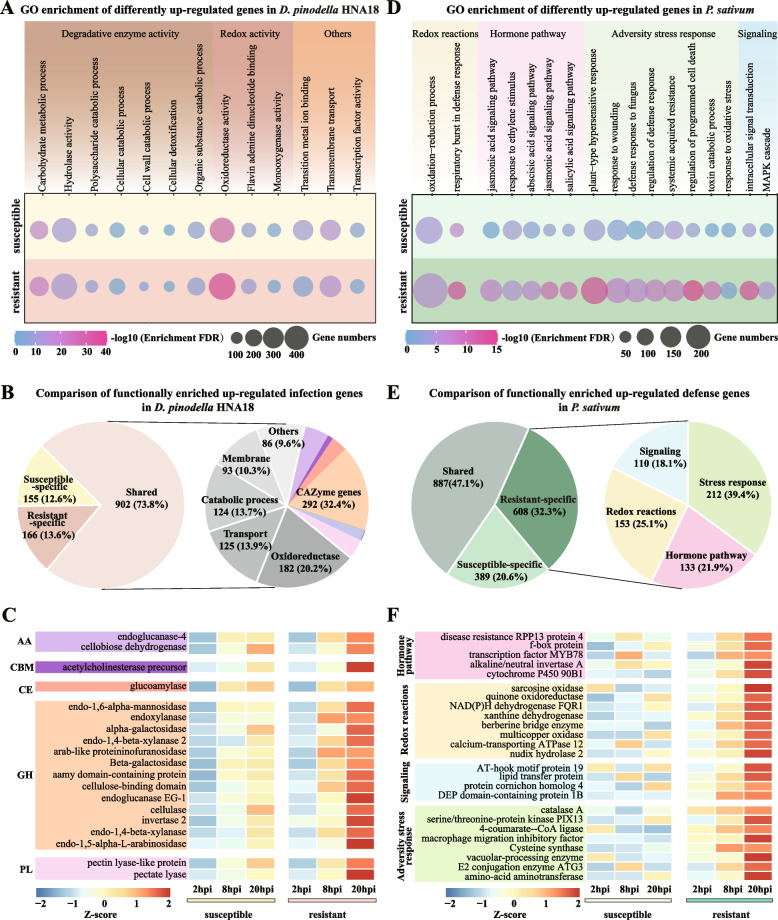
Comparative transcriptome analysis of *D. pinodella* HNA18 and disease-susceptible and disease-resistant pea varieties. **A** Gene ontology enrichment analysis of differentially upregulated genes in *D. pinodella* HNA18 when infecting disease-susceptible and disease-resistant peas during the entire infection process. **B** Comparison of functionally enriched upregulated infection genes in *D. pinodella* HNA18 for disease-susceptible and disease-resistant pea varieties. **C** Heatmap of the expression of 19 important infection genes in *D. pinodella* HNA18 during the entire infection process. **D** Gene ontology enrichment analysis of differentially upregulated genes of disease-susceptible and disease-resistant peas in response to *D. pinodella* HNA18 infection during the entire infection process. **E** Comparison of functionally enriched upregulated defense genes in disease-susceptible and disease-resistant pea varieties in response to *D. pinodella* HNA18 infection. **F** Heatmap of the expression of 25 important defense genes in disease-susceptible and disease-resistant pea varieties in response to *D. pinodella* HNA18 infection during the infection process

Functional enrichment analysis of genes significantly upregulated in *D. pinodella* HNA18 when infecting disease-susceptible (2,144 fungal genes) and disease-resistant peas (2,691 fungal genes) revealed that genes with degradative and redox activity are overrepresented. More specifically, enrichment analysis was separately conducted on the 2,144 and 2,691 differentially upregulated genes in *D. pinodella* HNA18 when infecting disease-susceptible and -resistant *P. sativum*, respectively. These analyses revealed that 1,068/2,144 (50%) and 1,057/2,691 (39%) of the differentially upregulated genes in *D. pinodella* HNA18 infecting disease-susceptible and disease-resistant pea varieties were functionally associated with degradative enzyme activity and redox activity (Fig. 5A). Among the genes associated with enriched functional categories, 902 (73.8%) were shared when *D. pinodella* HNA18 infected both disease-susceptible and disease-resistant pea varieties, whereas 155 (12.6%) and 166 (13.6%) were specific for disease-susceptible and disease-resistant hosts, respectively (Fig. 5B). Of the 902 genes, 292 (32.4%) were CAZymes that are associated with degrading plant polysaccharides [28, 29]. The expression profile of 19/292 (6.5%) CAZymes displayed increased expression as disease progressed (Fig. 5C); expression levels were often higher when infecting the disease-resistant, rather than disease-susceptible, host (Fig. 5C and Supplementary Table 5).

### Transcriptomics provides clues into different mechanisms of combating disease in pea varieties

To determine how the host transcriptional response changes as disease progresses, we conducted differential gene expression analysis in the disease-susceptible and disease-resistant pea varieties. These analyses revealed that the disease-susceptible pea differentially expressed 6,247 genes (3,275 upregulated and 2,972 downregulated) at 8 hpi compared to 2 hpi and 5,647 genes (2,882 upregulated and 2,765 downregulated) at 20 hpi compared to 2 hpi; the disease-resistant pea differentially expressed 5,573 genes (3,100 upregulated and 2,473 downregulated) at 8 hpi compared to 2 hpi and 5,268 genes (2,954 upregulated and 2,314 downregulated) at 20 hpi compared to 2 hpi (Supplementary Figure 3 and Supplementary Table 4). These results suggest that disease-susceptible and disease-resistant pea varieties do not vastly differ in the total number of differentially expressed defense genes.

To determine whether disease-resistant and disease-susceptible pea varieties differed in their transcriptional response to infection across the later stages of disease, we conducted functional enrichment analysis among the 4,031 and 3,939 uniquely upregulated genes in disease-susceptible and *disease*-resistant pea varieties, respectively. The number of enriched upregulated genes (1,495) in disease-resistant *P. sativum* 086 was higher than that of enriched upregulated genes (1,276) in disease-susceptible *P. sativum* 043 for functional categories related to redox reactions, hormone pathway, adversity stress response, and signaling (Fig. 5D). Of these, 887 (47.1%) disease resistance genes were shared between disease-resistant and disease-susceptible pea varieties. Among 608 functionally enriched upregulated defense genes specific to the disease-resistant *P. sativum* 086 (Fig. 5E), 25 (4%) increased throughout disease progression; in contrast, these genes lacked any obvious pattern in the disease-susceptible pea (Fig. 5F and Supplementary Table 6). These results underscore unique and shared transcriptional responses to *D. pinodella* HNA18 infection in disease-resistant and disease-susceptible pea varieties.

Collectively, the transcriptome profiles and functional enrichment analysis of upregulated infection genes in *D. pinodella* with respect to disease-susceptible and disease-resistant *P. sativum* varieties revealed that pathogenic fungus mobilized a similar set of infection genes to attack the disease-susceptible and disease-resistant pea varieties, but the timing and intensity of these infection genes were different. In contrast, the transcriptome profiles and functional enrichment analysis of upregulated defense genes in the disease-susceptible and disease-resistant *P. sativum* varieties in response to the infection of *D. pinodella* HNA18 showed that disease-susceptible and disease-resistant pea varieties mobilized similar types of defense genes, but the disease-resistant pea used a higher number of defense genes than the disease-susceptible pea.

## Discussion

Ascochyta blight is a devastating disease of peas. To date, at least seven fungal species have been identified as pathogens of pea ascochyta blight, including *Ascochyta pisi*, *Ascochyta koolunga*, *Ascochyta pinodes*, *Ascochyta pinodella*, *Phoma herbarum*, *Phoma glomerata*, and *Boeremia exigua var. exigua* [5, 6, 18, 30]. However, the pathogens in different pea-growing regions are distinct. For example, in Canadian prairies and southern Australia, *A. pinodes* and *P. koolunga* are the major pathogens of the *ascocoyta* blight complex, whereas *A. pisi* is the major pathogenic agent in North America, western Canada, and southern France [7, 31, 32]. In China, *A. pinodes* and *A. pisi* have been reported to be able to cause pea ascocoyta blight [33]. Here, we found that *Didymella pinodella* is another causal agent of pea ascocoyta blight. To our knowledge, this is the first report of *D. pinodella* as a pathogen of pea ascocoyta blight in the field.

Studies of interactions between pathogenic fungi and host plants largely vary. Some studies have focused on either interactions between different pathogenic fungi and the same host plant or interactions between one pathogenic fungus and different host plant species [8, 9, 11, 34]. However, few studies have focused on the mode of interactions between the same pathogenic fungus and different resistant species of the same host plant. This leaves us with little knowledge about the attack strategies of pathogenic fungi and the defense strategies of disease-susceptible and disease-resistant pea varieties during infection processes. By sequencing and analyzing the transcriptome data of the pathogenic fungus *D. pinodella* HNA18 and disease-susceptible and disease-resistant pea varieties during infection, we found that the pathogenic fungus mobilized a similar set of infection genes to attack disease-susceptible and disease-resistant pea varieties, but the timing and intensity of these infection genes varied. Conversely, disease-susceptible and disease-resistant peas mobilized similar types of defense genes, while disease-resistant peas used a higher number of defense genes than disease-susceptible peas. This study reveals the pattern of interaction between pathogen infection and the defense of different resistant varieties of the host plant pea.

The temporal transcriptome design of the infection process of *D. pinodella* HNA18 and susceptible and resistant pea varieties not only provides a very practical tool to study the temporal expression dynamics of pathogenic fungal-host plant interactions but also identifies an important set of infection genes in *D. pinodella* HNA18 and of defense genes in pea. For example, an endo-β-1,4-xylanase (*xyn11A*), which promotes the virulence of pathogenic fungi through its necrotrophic properties in *Botrytis cinerea*, was considered to be an important virulence factor [35]. An extracellular endogenous-1,4-β-glucanase (*PlEGL1*) identified in *Pyrenochaeta lycopersici* can help the fungus to disrupt the host cell wall [36]. In the list of our pea defense genes, *RPP13* is a resistance gene that exhibits a high level of polymorphism determining the resistance of *Arabidopsis parasitica* and *Hyaloperonospora parasitica* [37, 38]. In addition, *Arabidopsis* berberine-bridging enzyme-like enzyme (*OGOX*) plays a crucial role in plant immunity, and *OGOX* overexpression resulted in increased resistance to infection by the pathogenic fungus *Botrytis cinerea* [39]. In addition to these previously reported infection genes in pathogenic fungi and defense genes in host plants, we also provide a fraction of less well-studied infection and defense genes, which might play an important role in controlling fungal disease and breeding pea-resistant varieties.

## Materials and methods

### Fungal isolation, morphological characteristics and pathogenicity testing

The HNA18 strain was isolated from infected pea leaves with typical symptoms of ascochyta blight at the Zhejiang province field station (30.25 N, 120.21E), China, in 2020. Infected leaves were cut into pieces and surface sterilized with a 30 s treatment in 70% ethanol followed by 15 min in sodium hypochlorite (10% active chlorine) and three subsequent washing steps with sterile water for at least 15 min each. Sterilized samples were placed onto potato dextrose agar (PDA) plates (200 g potato, 20 g glucose, 15 g agar, and 1 L water) supplemented with chloramphenicol (10 μg/ml) and kanamycin (25 μg/ml) and incubated at 25 °C for 3 days. After incubation, the edges of fungal colonies were cut out and transferred to new plates for purification. Single conidium-derived isolates were prepared and stored at 4 °C for further study.

Subsequently, HNA18 was purified using a single spore and cultured on complete medium (CM) for morphological identification. The production of pycnidia was studied on 1/2 CM plates. After 10 days of incubation at 25 °C, the conidia were stained with calcofluor white (a fluorescent dye that binds to cell walls) (catalog no. 18909; Sigma‒Aldrich, St. Louis, MO) and visualized using the Leica (Wetzlar, Hesse-Darmstadt, Germany) TCS SP5 imaging system.

For the pathogenicity test, the pea leaves, stems, and pods were sterilized and inoculated with mycelial discs (0.5 cm in diameter) of HNA18. Inoculated samples were incubated in a growth chamber at 25 °C with 100% humidity and a 12-h photoperiod. The disease symptoms of inoculated samples were imaged from 3 days postinoculation.

### DNA extraction and genome sequencing

The HNA18 strain was grown for three days in potato dextrose liquid media at approx. 22℃ with shaking (150 rpm). For Illumina sequencing, DNA was prepared using a standard CTAB extraction method. RNA was removed by incubation with DNase-free RNase A, and DNA was resuspended in TE buffer (10 mM Tris-HCl 1 mM EDTA, pH 8). The DNA concentration was determined by a NanoDrop spectrophotometer (Thermo-Fisher Scientific, Waltham, MA, USA). For PacBio sequencing, maxi-prep DNA was prepared using a modified method from Xin and Chen [40]. Genome sequencing was performed by Novogene Co., Ltd., using the 150-bp paired-end Illumina HiSeq and single-molecule, real-time (SMRT) PacBio sequencing platforms. We used PacBio sequencing techniques to generate 13.7 GB reads.

### Reference genome assembly and genome quality evaluation

Genome size was first evaluated briefly using the k-mer count in Jellyfish v2.2.10 [41]. PacBio SMRT reads were assembled using CANU v2.2 [42], which first conducted self-correction for the raw data and then assembled the corrected reads. The resulting assembly sequences were corrected using Illumina reads via NextPolish v1.3 [43]. The mitochondrial genome was reassembled from whole genome data using NOVOPlasty v4.3.1 [44]. The statistics for the genome assembly of HNA18 were calculated using the software QUAST v 4.6.2 [45]. Telomere sequences were examined based on the presence of “TTAGGG” tandem repeat sequences at contig ends, following previous studies [5, 6, 18, 19].

To assess the quality of the genome assembly, we used BUSCO v5.2.2 [46] to identify conserved genes within the “pleosporales_odb10” databases [47].

### Genome annotation

Genome annotation of the assembly was performed using the BRAKER2 pipeline v2.1.5 [48]. RNA-seq reads were used for annotation. RNA-seq sequencing was performed on an Illumina HiSeq 2000 machine at Novogene (Beijing, China). After removing low-quality sequences, the clean RNA-seq reads were aligned to genomes with STAR (Spliced Transcripts Alignment to a Reference) v2.7.6 [49] in 2-pass mode. Gene predictions were carried out using de novo RNA-seq evidence using Augustus v3.3.3 [50] and GeneMark-ET v4.38 [51]. Genome annotations were assessed for completeness using BUSCO v5.2.2 [46] (–m prot) “pleosporales_odb10” databases [47]. Gene function annotation was performed with eggNOG-mapper v.5.0 [52].

### Phylogenetic analysis

Seven publicly available *Didymella* species genomes were retrieved from NCBI, and their genome qualities were evaluated using BUSCO v5.2.2 [46]. Here, we used 5,699 single-copy BUSCO genes that are present in at least four of eight *Didymella* species to build the phylogenetic tree. Each BUSCO gene was aligned with MAFFT v7.471 [53] with options “--auto”. Ambiguously aligned regions were removed using the ‘‘gappyout’’ option in trimAl v1.4 [54]. The nucleotide sequence alignments of these 5,699 BUSCO genes were then concatenated into the full data matrix with PhyKIT v1.2.1 [55]. The maximum likelihood phylogenetic analyses were performed using IQ-TREE, version 1.6.12 under the GTR+G4+F model [56].

### Gene family of eight *Didymella* species

Homology clustering of all proteomic sequences of eight *Didymella* species was implemented in OrthoFinder v2.5.2. with the default parameters [57]. Gene families shared by all species are defined as core protein families, but gene families present in individual species are defined as species-specific ones.

### Prediction of CAZymes

CAZymes were predicted using the CAZymes database [58, 59]. Each *Didymella* protein was compared with proteins listed in the CAZymes database using HMMER v3 [60]. The proteins with ≥ 50% identity in the CAZymes database were assigned to the same family/subfamily. Proteins with less than 50% identity were manually inspected.

### Prediction and analysis of secondary metabolite gene clusters

The secondary metabolite gene clusters (SMGCs) of the eight *Didymella* genomes were predicted using the AntiSMASH v5.0 tool [61]. Backbone proteins are defined as proteins with the annotations PKS(-like), NRPS(-like), hybrid, and TC. The analysis of the SMGC families was based on the Pfam domains (http://pfam.xfam.org/). Big-SCAPE v1.1.0 [62] was used to explore the diversity of the biosynthetic gene clusters (BGCs) for the SMGCs.

### RNA sequencing and transcriptome analysis

Mycelia of *D. pinodella* HNA18 for RNA sequencing were obtained from 18 samples after infecting disease-susceptible *P. sativum* 043 and disease-resistant *P. sativum* 086. Mycelia were isolated from leaves at different time points (2 h, 8 h, 20 h). For plant samples, we inoculated leaves of the susceptible *P. sativum* 043 and disease-resistant *P. sativum* 086 in response to *D. pinodella* HNA18. Pea leaves were also collected for RNA-seq at 2 h, 8 h and 20 h postinoculation (hpi). There were three biological replicates for each treatment. For RNA-seq data, library construction and sequencing were performed on an Illumina HiSeq2000 (pair ends) (Fig. 4A).

Raw RNA-seq reads were removed of low-quality reads and adapter sequences using Trimmomatic v0.39 [63] with default parameters. Clean reads were mapped to the reference *D. pinodella* HNA18 genome and *P. sativum* v1a genome using STAR v2.7.6a [49]. The read number for each gene was counted by featureCounts v1.6.0 [64], and the resulting transcript count tables were subjected to differential expression analysis using the R packages edgeR v3.360 [65] and limma v3.50.0 [66]. Transcripts with an adjusted *P* value of ≤ 0.05 and log2-fold change of ≥ 1 or ≤ -1 were determined to be differentially expressed genes. The unit for the expression level of each protein-coding gene is fragments per kilobase of transcript per million mapped reads (FPKM). Gene Ontology (GO) enrichment analysis of differentially up- or downregulated genes was conducted using ShinyGO v0.75 (http://bioinformatics.sdstate.edu/go/) [67]. All statistical analyses were performed in R v. 3.6.3 (R core team 2021).

## Supplementary Information

**Additional file 1: Supplementary Figure 1.** The composition of secondary metabolic gene clusters (SMGCs) in eight *Didymella *species. Predicted secondary metabolite genes for each species were divided by the backbone enzyme. NRPS: nonribosomal peptide synthetase, PKS: polyketide synthase, HYBRID: a backbone gene containing domains from NRPS and PKS backbones, NRPS-like: nonribosomal peptide synthetase-like, TC: terpene cyclase.

**Additional file 2: Supplementary Figure 2.** Analysis of differentially expressed genes in *D. pinodella* HNA18 infecting disease-susceptible (left panel) and disease-resistant (right panel) pea varieties. (A) Volcano plots of differentially expressed genes (DEGs) in *D. pinodella* HNA18 infecting disease-susceptible pea 043 and disease-resistant pea 086 in 8 hpi vs 2 hpi analysis, respectively. (B) Volcano plots of differentially expressed genes (DEGs) in *D. pinodella* HNA18 infecting disease-susceptible pea 043 and disease-resistant pea 086 in 20 hpi vs 2 hpi analysis, respectively.

**Additional file 3: Supplementary Figure 3.** Analysis of differentially expressed genes in disease-susceptible (left panel) and disease-resistant (right panel) pea varieties in response to *D. pinodella *HNA18 infection. (A) Volcano plots of differentially expressed genes (DEGs) in disease-susceptible pea 043 and disease-resistant pea 086 in response to the infection of *D. pinodella *HNA18 in 8 hpi vs 2 hpi analysis. (B) Volcano plots of differentially expressed genes (DEGs) in disease-susceptible pea 043 and disease-resistant pea 086 in response to the infection of *D. pinodella *HNA18 in 20 hpi vs 2 hpi analysis.

**Additional file 4: Supplementary Table 1.** Genome information and gene family analysis of eight *Didymella* species. **Supplementary Table 2.** Information on RNA-seq data in this study. **Supplementary Table 3.** List of differentially expressed genes in *D. pinodella* HNA18 infecting disease-susceptible and disease-resistant pea varieties. **Supplementary Table 4.** List of differentially expressed genes in disease-susceptible and disease-resistant pea varieties in response to *D. pinodella* HNA18 infection. **Supplementary Table 5.** List of important infection genes and their expression in *D. pinodella* HNA18 infecting disease-susceptible and disease-resistant pea varieties. **Supplementary Table 6.** List of important defense genes and their expression in disease-resistant pea varieties in response to *D. pinodella* HNA18 infection.

## Data Availability

The genome of *Didymella pinodella* HNA18 has been deposited at the National Genomics Data Center (NGDC) under accession number GWHBOVH00000000. Illumina sequencing data have been submitted to the National Center for Biotechnology Information (NCBI) Sequence Read Archive (SRA) under Bio-Project PRJNA887843 and PRJNA887921.
