# Supplementary material for: Temporal transcriptomics provides insights into host‒pathogen interactions: a case study of Didymella pinodella and disease-resistant and disease-susceptible pea varieties
Source: Crop Health. 2023 Aug 10;1(1):5. doi: 10.1007/s44297-023-00005-w (PMC12825973; doi:10.1007/s44297-023-00005-w)
Supplement: Supplementary file 1 — Additional file 1: Supplementary Figure 1. The composition of secondary metabolic gene clusters (SMGCs) in eight Didymella species. Predicted secondary metabolite genes for each species were divided by the backbone enzyme. NRPS: nonribosomal peptide synthetase, PKS: polyketide synthase, HYBRID: a backbone gene containing domains from NRPS and PKS backbones, NRPS-like: nonribosomal peptide synthetase-like, TC: terpene cyclase. [file 44297_2023_5_MOESM1_ESM.pdf]

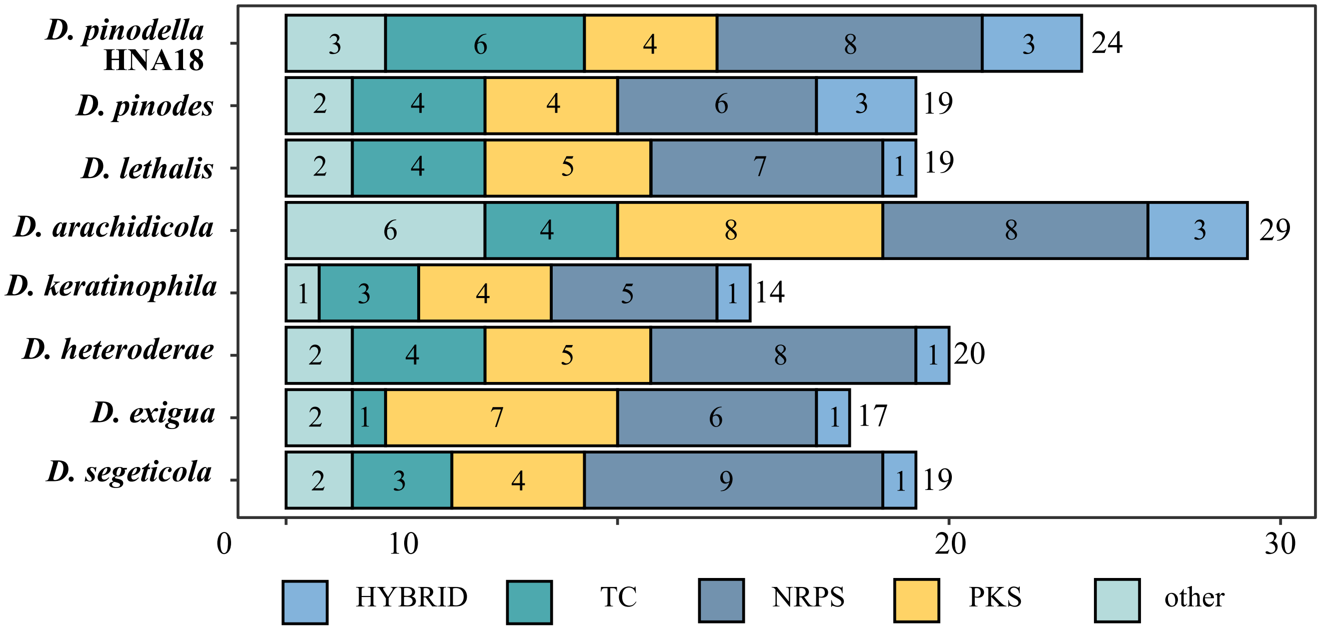

Supplementary Figure 1. The composition of secondary metabolic gene clusters (SMGCs) in eight *Didymella* species. Predicted secondary metabolite genes for each species were divided by the backbone enzyme. NRPS: non-ribosomal peptide synthetase, PKS: polyketide synthase, HYBRID: a backbone gene containing domains from NRPS and PKS backbones, NRPS-like: non-ribosomal peptide synthetase like, TC: terpene cyclase.
