# Supplementary material for: Temporal transcriptomics provides insights into host‒pathogen interactions: a case study of Didymella pinodella and disease-resistant and disease-susceptible pea varieties
Source: Crop Health. 2023 Aug 10;1(1):5. doi: 10.1007/s44297-023-00005-w (PMC12825973; doi:10.1007/s44297-023-00005-w)
Supplement: Supplementary file 2 — Additional file 2: Supplementary Figure 2. Analysis of differentially expressed genes in D. pinodella HNA18 infecting disease-susceptible (left panel) and disease-resistant (right panel) pea varieties. (A) Volcano plots of differentially expressed genes (DEGs) in D. pinodella HNA18 infecting disease-susceptible pea 043 and disease-resistant pea 086 in 8 hpi vs 2 hpi analysis, respectively. (B) Volcano plots of differentially expressed genes (DEGs) in D. pinodella HNA18 infecting disease-susceptible pea 043 and disease-resistant pea 086 in 20 hpi vs 2 hpi analysis, respectively. [file 44297_2023_5_MOESM2_ESM.pdf]

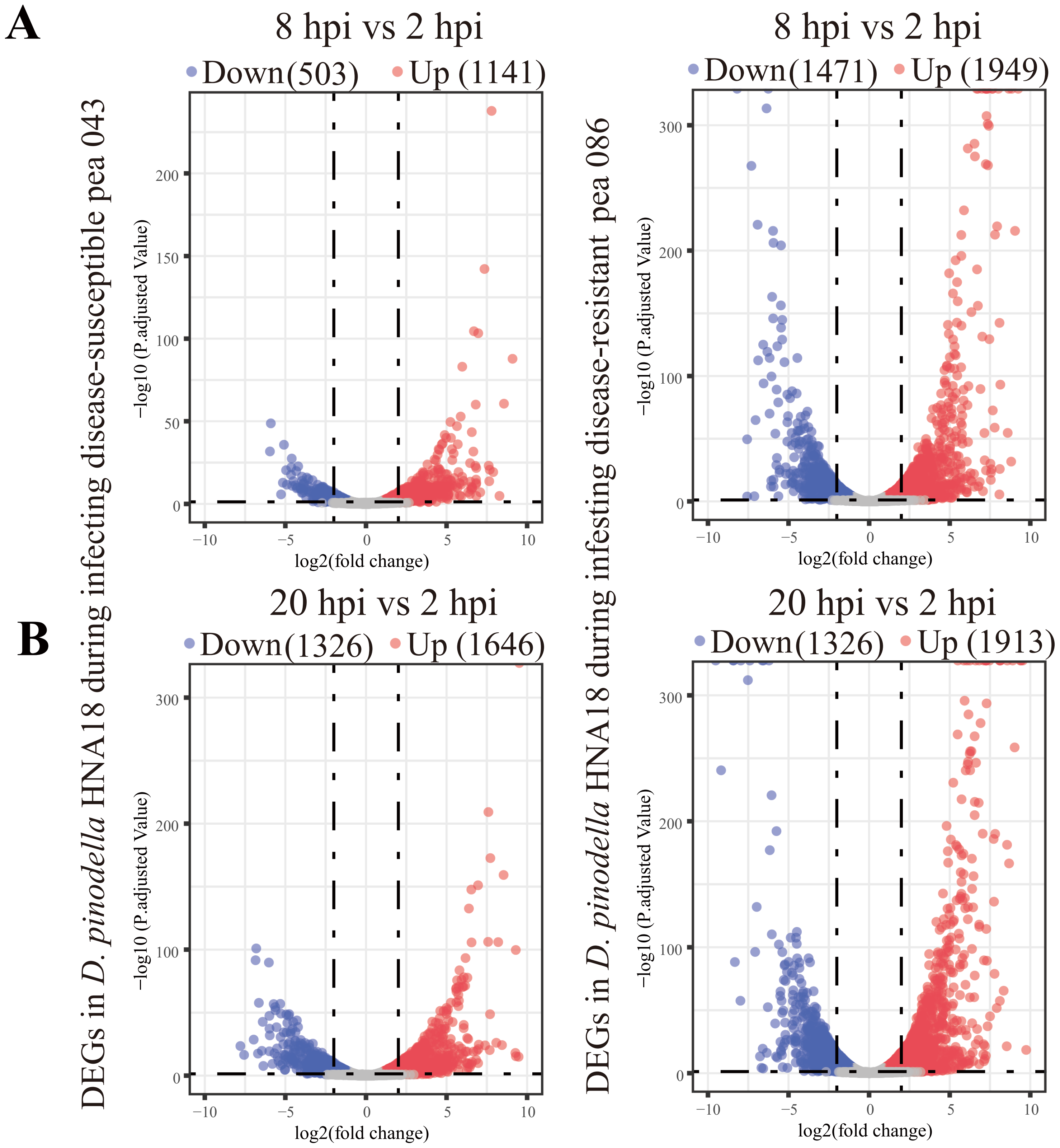

Supplementary Figure 2. Analysis of differentially expressed genes in *D. pinodella* HNA18 infecting disease-susceptible (left panel) and disease-resistant (right panel) pea varieties. (A) Volcano plots of differentially expressed genes (DEGs) in ***D. pinodella*** HNA18 infecting disease-susceptible pea 043 and disease-resistant pea 086 in 8 hpi vs 2 hpi analysis, respectively. (B) Volcano plots of differentially expressed genes (DEGs) in ***D. pinodella*** HNA18 infecting disease-susceptible pea 043 and disease-resistant pea 086 in 20 hpi vs 2 hpi analysis, respectively.
