# Supplementary material for: Temporal transcriptomics provides insights into host‒pathogen interactions: a case study of Didymella pinodella and disease-resistant and disease-susceptible pea varieties
Source: Crop Health. 2023 Aug 10;1(1):5. doi: 10.1007/s44297-023-00005-w (PMC12825973; doi:10.1007/s44297-023-00005-w)
Supplement: Supplementary file 3 — Additional file 3: Supplementary Figure 3. Analysis of differentially expressed genes in disease-susceptible (left panel) and disease-resistant (right panel) pea varieties in response to D. pinodella HNA18 infection. (A) Volcano plots of differentially expressed genes (DEGs) in disease-susceptible pea 043 and disease-resistant pea 086 in response to the infection of D. pinodella HNA18 in 8 hpi vs 2 hpi analysis. (B) Volcano plots of differentially expressed genes (DEGs) in disease-susceptible pea 043 and disease-resistant pea 086 in response to the infection of D. pinodella HNA18 in 20 hpi vs 2 hpi analysis. [file 44297_2023_5_MOESM3_ESM.pdf]

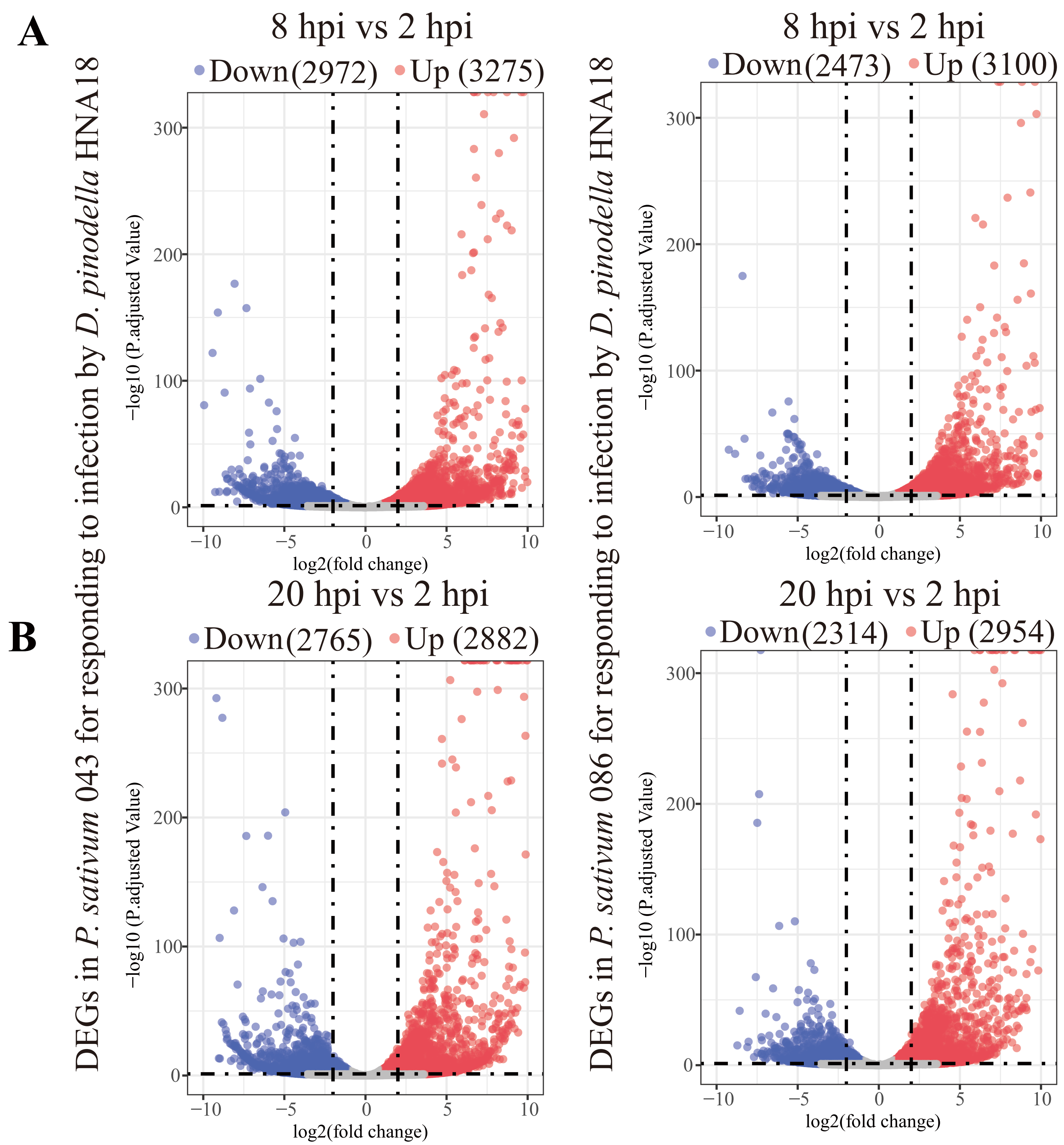

Supplementary Figure 3. Analysis of differentially expressed genes in disease-susceptible (left panel) and disease-resistant (right panel) pea varieties in response to the infection of *D. pinodella* HNA18. (A) Volcano plots of differentially expressed genes (DEGs) in disease-susceptible pea 043 and disease-resistant pea 086 in response to the infection of *D. pinodella* HNA18 in 8 hpi vs 2 hpi analysis, respectively. (B) Volcano plots of differentially expressed genes (DEGs) in disease-susceptible pea 043 and disease-resistant pea 086 in response to the infection of *D. pinodella* HNA18 in 20 hpi vs 2 hpi analysis, respectively.
